# The Crucial Role of Molecular Biology in Cancer Therapy: A Comprehensive Review

**DOI:** 10.7759/cureus.52246

**Published:** 2024-01-14

**Authors:** Prisha Gulati, Chandra Veer Singh

**Affiliations:** 1 Medicine and Surgery, Jawaharlal Nehru Medical College, Datta Meghe Institute of Higher Education and Research, Wardha, IND; 2 Otolaryngology - Head and Neck Surgery, Jawaharlal Nehru Medical College, Datta Meghe Institute of Higher Education and Research, Wardha, IND

**Keywords:** crispr, tumor, viral infection, mutagenesis, proto-oncogenes, oncogenes, cancer

## Abstract

Molecular biology shines a light of hope amid the complex terrain of cancer, bringing revolutionary approaches to cancer treatment. Instead of providing a synopsis, this review presents an engaging story that sheds light on the genetic nuances controlling the course of cancer. This review goes beyond just listing genetic alterations to examine the complex interactions that lead to oncogene activation, exploring particular triggers such as viral infections or proto-oncogene mutations. A comprehensive grasp of the significant influence of oncogenes is possible through the classification and clarification of their function in various types of cancer. Furthermore, the role of tumor suppressor genes in controlling cell division and preventing tumor growth is fully explained, providing concrete examples and case studies to ground the conversation and create a stronger story. This study highlights the practical applications of molecular biology and provides a comprehensive overview of various detection and treatment modalities. It emphasizes the effectiveness of RNA analysis, immunohistochemistry, and next-generation sequencing (NGS) in cancer diagnosis and prognosis prediction. Examples include the individualized classification of breast cancers through RNA profiling, the use of NGS to identify actionable mutations such as epidermal growth factor receptor and anaplastic lymphoma kinase in lung cancer, and the use of immunohistochemical staining for proteins such as Kirsten rat sarcoma viral oncogene to guide treatment decisions in colorectal cancer. This paper carefully examines how molecular biology is essential to creating new strategies to fight this difficult and widespread illness. It highlights the exciting array of available therapeutic approaches, offering concrete instances of how clustered regularly interspaced short palindromic repeats (CRISPR) and CRISPR-associated protein 9 (CRISPR-Cas9), targeted pharmaceuticals, immunotherapy, and treatments that induce apoptosis are driving a paradigm shift in cancer care. The revolutionary CRISPR-Cas9 system takes center stage, showcasing how precise gene editing could transform cancer therapy. This study concludes by fervently highlighting the critical role that molecular biology plays in reducing the complexity of cancer and changing the treatment landscape. It lists accomplishments but also thoughtfully examines cases and findings that progress our search for more precisely customized and effective cancer therapies.

## Introduction and background

Cellular genetic alterations lead to the condition known as cancer. Oncogenes must be activated for cancer to develop, which can occur in one of two ways. Oncogenes can be triggered by tumor viruses that attack cells or transform from normal genes (known as proto-oncogenes) to become oncogenes. For example, the transformation of proto-oncogenes, such as SRC evolving into v-Src oncogene due to mutation, and ABL proto-oncogenes forming the BCR-ABL oncogene through gene fusion. One cell’s oncogenic change can start the development of a tumor. Tumor formation is triggered by oncogenic alterations, which are caused by gene mutations such as proto-oncogenes, causing normal cells to turn malignant. The fact that cancer cells can spread from the main tumor to other bodily areas, making therapy more difficult and frequently producing less-than-ideal results, is the significance of metastasis. Targeted therapies that aim to interfere with the molecular course of cancer improve patient outcomes through improved diagnosis and treatment strategies are informed by research into these mechanisms. Studies have found that the Rous sarcoma virus might spread malignancy between animals in the case of solid tumors such as sarcomas. Rous won the Nobel Prize for this endeavor. Later research revealed that tumors could also form without viral DNA [1]. The Rous sarcoma virus was the subject of Peyton Rous’s pioneering research in the early 1900s. He found this virus that causes cancer in hens, and in 1966, he was awarded the Nobel Prize in Physiology or Medicine for his pioneering research. A turning point in the study of cancer was reached when Rous discovered a virus that could cause tumors, providing insights into the viral causes of some types of the disease. This finding prompted more research into oncogenic viruses, which profoundly affected our comprehension of the molecular mechanisms behind cancer and opened up new avenues for virology and cancer biology studies. The widespread effect of cancer, which affects millions of lives and is a major cause of illness and mortality globally, highlights the urgent need for novel approaches to its understanding and treatment.

## Review

Methodology

**Figure 1:**
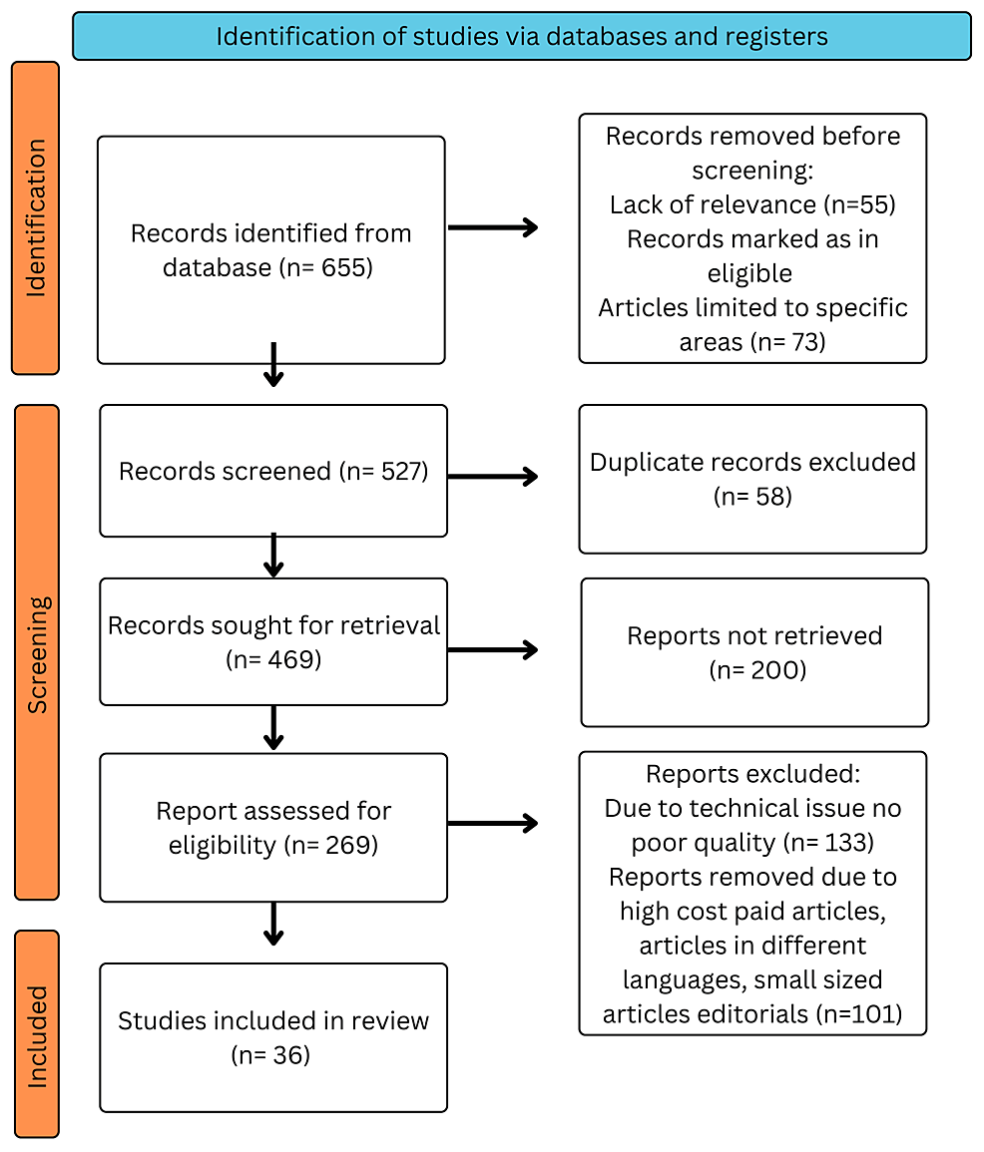
Preferred Reporting Items for Systematic Reviews and Meta-Analyses flowchart.

We performed a comprehensive search using PubMed, MEDLINE, Embase, Google Scholar, and ResearchGate. A search of the English-language literature was done. It was also the subject of a different search. The query terms were “cancer” OR “oncogenes”; “proto-oncogenes” OR “mutagenesis”; “viral infection” OR “tumour”; “CRISPR” OR “tumour suppressor genes”; “treatment.” The articles in this review met the following requirements: studies conducted exclusively on advancements in understanding molecular biology in cancer treatment and new treatment interventions and studies conducted in English from 1975 to 2016. Figure 1 highlights the Preferred Reporting Items for Systematic Reviews and Meta-Analyses (PRISMA) method used in this study.

The article explores the connection between carcinogens and mutagens, highlighting the fact that agents that might cause cancer frequently cause mutations in DNA. These mutations can change healthy genes, which can result in the development of malignant cells. Research on molecular biology has produced important information in favor of this view [2]. It is imperative, therefore, to recognize possible gaps in the literature, taking into account sample limits or methodological restrictions that may affect the wider relevance of the conclusions. In addition, evaluating the applicability and currency of these discoveries is crucial, considering the dynamic nature of scientific knowledge. Tumor suppressor gene loss or degradation can trigger oncogenes and interfere with the regulation of cell proliferation, therefore, hastening the development of cancer. Both of these pathways are frequently involved in tumor development [3].

Oncogenes in the genesis of cancer

Oncogenes are genes that have the potential to develop into cancer and are crucial in the development of many different forms of cancer. A significant turning point in the study of cancer was the 1970 identification of the SRC oncogene in a chicken retrovirus. Proto-oncogenes, ordinarily normal genes, can develop unregulated states due to mutations, which can cause unchecked cell proliferation and, eventually, cancer [4]. Because of the dominant nature of the oncogenic allele, only one is required at the genomic level to change the way a gene functions normally. Oncogenes can come from the body (cellular) or viruses (viral) [3]. Some proto-oncogenes can change their function and become oncogenes by duplication, addition, insertion, deletion, or chromosomal rearrangement. Tumor development may result from the unregulated overexpression of the protein brought on by these mutations. These mutations may result from viral infections, radiation or chemical exposure, damage, or illness, among other external, internal, or a combination of sources [4]. Although viral infections rarely activate oncogenes in animals, they are essential to understanding how oncogenes work.

Viral infection

Retroviruses and DNA viruses are both capable of causing viral illnesses. These viruses either introduce oncogenes into the host chromosome, interfere with the transcription factors or regulators of proto-oncogenes, or insert homologous sequences similar to the host’s typical proto-oncogenes to infect the host. For instance, a retrovirus that carries the SRC oncogene can infect the host by integrating its viral chromosome into the host chromosome, splitting into viral offspring, and infecting neighboring cells. This causes unchecked cell growth and normal cellular gene overexpression, ultimately leading to cancer [5].

Oncogene types and classification

Not all oncogenes may fall neatly into these categories. It is crucial to remember that the categorization of oncogenes into five types is based on the kind of protein product generated by mutation or dysregulation of proto-oncogenes. The five types include (1) growth factors and their receptors, (2) intracellular signal transducers, (3) transcription factors, (4) cell cycle regulators, and (5) apoptosis regulators. However, these classifications offer a helpful foundation for comprehending the variety of oncogenic mutations that can result in cancer [6,7]. Along with the previously mentioned examples, it should be noted that mutations in additional oncogenes, such as MYC and BCL2, have been linked to various cancers. MYC, a transcription factor that regulates the expression of multiple genes involved in cell growth and proliferation, is typically overexpressed in Burkitt lymphomas. BCL2 is an anti-apoptotic protein that prevents intended cell death, and its overexpression has been linked to several cancers, including lymphomas, leukemias, and breast cancer [8]. Overall, the variety of mutations that may activate oncogenes shows the complexity of cancer development and the need for ongoing research into the underlying molecular pathways causing this illness.

The function of oncogenes in cancer treatment

Drugs and gene treatments that target oncogenes are used to treat cancer. Various techniques, such as inhibition, regulation, arrest, or senescence of their genes, are used to target various oncogenes. For instance, BCR-ABL is treated with imatinib (commonly referred to as Gleevec), an ABL kinase inhibitor. Bevacizumab or sorafenib are medications used to target vascular endothelial growth factor oncogenes, whereas medicines such as gefitinib (Iressa), erlotinib (Tarceva), and others are used to target epidermal growth factor receptors. The B-Raf oncogene can also be downregulated or inhibited by sorafenib. Combinations of chemotherapy may also be used to treat oncogenic tumors by preventing the growth of oncogenes or by suppressing signaling oncoproteins in several signaling pathways. Drug targets for non-kinase oncogenes such as Myc and Ras are more difficult to find [8].

Tumor suppressor genes in cancer

Inhibiting cellular proliferation and halting the growth of tumors are largely accomplished owing to tumor suppressor genes. The inactivation of tumor suppressor genes in many forms of tumors eliminates their negative control on cell growth, resulting in aberrant cell proliferation and the emergence of cancer. Mutations known as loss of function in tumor suppressor genes render them incapable of inhibiting cell growth. One copy of a tumor suppressor gene is normally adequate to regulate cell proliferation, but both copies are required to stimulate tumor formation. These changes have a recessive effect [9].

Role of tumor suppressor genes in the context of cancer

The crucial roles identified to be performed by the protein products of tumor-suppressing genes include the formation of checkpoint-control proteins to stop the cycle in case of DNA damage or chromosomal abnormalities. Enzymes involved also help in DNA repair and apoptosis. These proteins also serve as hormone receptors to restrain cellular growth and proliferation. Along with all these functions, intracellular proteins control or impede progression through a particular cell cycle stage [10].

Tumor suppressor gene function in cancer gene for Wilms tumor 1

Some genes that suppress tumors function as transcriptional regulators. For instance, the WT1 gene produces a repressor protein that prevents the transcription of a large number of growth factor-inducible genes. In children’s kidney tumors called Wilms tumors, WT1 is rendered inactive. The WT1 gene overexpression in Wilms tumors targets insulin-like growth factor II, which aids in aberrant cell proliferation [11].

Retinoblastoma and INK4 genes

The entry into and exit from particular phases of the cell cycle are governed by tumor suppressor genes, which play a significant role in controlling cell cycle progression. The retinoblastoma (Rb) gene and the INK4 gene are two examples of these genes. Retinoblastoma, an eye tumor, can arise as a result of mutations in these genes. The Cdk2 and cyclin D complexes regulate the entry through the constraint point of the cell cycle in normal cells by phosphorylating and inactivating pRb, and pRb regulates the entry through the constraint point of the cell cycle in the G1 phase by suppressing the transcription of genes involved in cell cycle progression. The Cdk inhibitor p16, which similarly controls passing past the restriction point, is encoded by the INK4 tumor suppressor gene. Rb is phosphorylated without control when INK4 is inactivated [10].

Tumor suppressor gene p53

Cell cycle progression and programmed cell death are tightly controlled by the tumor suppressor gene p53. When DNA is damaged, p53 can stop the cell cycle to give time for DNA repair or, if the damage is irreversible, to cause programmed cell death (apoptosis). This is accomplished by turning on a variety of genes that regulate and govern the cell cycle. However, changes in the p53 gene can prevent it from acting as a tumor suppressor, which can cause unchecked cell growth and ineffective DNA repair. p53 mutations are thought to be the genetic changes that occur in human tumors most frequently, occurring in 50% or even more of instances [12].

Breast cancer-1 and 2 genes

Breast cancer family risk is associated with the breast cancer-1 and 2 genes. The breast cancer-1 gene has 21 exons and 100 kb of DNA. Similar to DNA-binding proteins, it features a zinc finger domain. Breast cancer-1 is a gene that inhibits cancer growth. Chromosome 13 contains the gene BRCA-2.

Tumor suppressor genes and their application

Tumor suppressor genes may be investigated at the DNA, mRNA, and protein levels in both healthy and malignant cells using various techniques. The likelihood of developing a particular type of cancer may be determined with the help of a heterozygosity test. Polymerase chain reaction (PCR) amplification, RNase protection assays, single-strand conformational polymorphism, and denaturing gel electrophoresis are methods for assessing genetic changes. Immunometric approaches may be utilized in tissue samples and tumor cell lysates to assess altered proteins, such as p53 [13,14].

Molecular pathology: diagnosis of cancer

The proper diagnosis of cancer patients is a key problem in their clinical care. Immunohistochemistry, immunofluorescence, and DNA and RNA analysis using in situ hybridization and fluorescent in situ hybridization are only a few of the technologies that have been developed for subtyping malignancies. The molecular biology methods used to subtype cancer specimens include Sanger sequencing, pyrosequencing, allele-specific PCR, snapshot assays, mass spectroscopy-based assays, and next-generation sequencing (NGS) using semiconductor or fluorescence detectors. The full variety of tumors has been revealed by NGS, and recurrent mutations that may be treated with novel medicines have been found. Such genomic-level studies will remain influential for a very long time to come [15,16].

Advancements in cancer treatment over the years

Numerous therapies and treatment methods have been created and are currently being used to treat cancer. These include apoptosis-inducing medications, targeted growth signal inhibition, surgery, radiation therapy, chemotherapy, hormonal therapy, immunotherapy, adjuvant therapy, nanotechnology, RNA expression and profiling, and the most recent development, CRISPR [17]. Oncolytic viruses can be employed in conjunction with chemotherapeutic drugs to kill cancer cells, as can gene replacement therapy or oncogene knockout. In this overview, we will go into further depth about a few of these therapy approaches [18].

Retroviral therapy for cancer

By delivering genes to mammalian cells, retroviruses (RVs) have been used as an alternate method to conventional cancer treatment. The RV most frequently employed for this purpose is the Moloney murine leukemia virus (MoMLV). RVs have been synthetically developed during the past 20 years to enable their application in transgenic animal development, reliable siRNA administration, and clinical gene therapy studies. Recent studies have shown that RVs can successfully cure severe immunodeficiency conditions in clinical trials. Despite the potential advantages, using RVs for gene therapy comes with inherent risks [19]. In a comparison experiment, two sets of vectors were created and evaluated; one was faulty and needed a helper retrovirus, while the other was innately replicative. The faulty group only transduced less than 1%, but the replicative viruses attained a transduction rate of over 85%. This study demonstrates the potential use of RVs in the creation of cancer gene therapy [20].

Insertional oncogenesis and retroviral tagging

Studying retroviral vector insertions, especially their potential to trigger oncogenesis, has attracted more attention in recent years. For many years, scientists have used viral insertion sites to find possible oncogenes and cancer signaling pathways. The reach of this strategy has, however, increased with the creation of new methods such as high-throughput PCR for insertion site cloning, the accessibility of animals that have undergone genetic modification, and the completion of the mouse genome project [21,22]. However, hundreds of common sites have been found by numerous researchers. In MoMLV-induced murine hematopoietic malignancies, these integration sites are typically linked to cancer genes [23-25]. Most insertions of retroviral vectors occur outside the coding domains of genes. Thus, tumour suppressor genes can be found in fewer than 10% of retroviral insertion sites (RISs). However, it is interesting to note that approximately 17%-18% of RISs target transcription factors [19].

Utilizing molecular biology techniques for cancer treatment strategies

In the past, target genes were knocked out or deactivated through homologous recombination to understand their activities. However, this method was time-consuming and labor-intensive, and it was not very effective at introducing constructs into the desired target site. This method was also linked to several serious mutagenesis effects [26].

Transcription activator-like effector nucleases (TALENs)

In that it requires DNA-binding domains and a nuclease domain for genome editing, the TALEN system is analogous to the ZFN system. TALENs, on the other hand, bind more strongly with their target sites and distinguish one nucleotide from another rather than recognizing a triplet of them [27,28]. Additionally, TALENs are simpler to design than ZFNs. Two specially designed TALENs are needed to detect the target gene’s DNA sequences on opposing strands to employ TALENs for cancer treatment. When the TALENs’ FokI nuclease cleavage domain dimerizes, it cleaves the target gene’s sequence and causes double-stranded DNA breaks. The target gene is altered by the end-joining DNA repair mechanism as it corrects the lesion caused by the DNA break because of the change in the reading frame. Using this method, pre-existing mutations can also be removed. TALEN technology is a powerful gene editing method for treating cancer cells as it can change complicated cancer genes and target any gene in the genome [29-32].

The CRISPR/Cas9 system: an effective tool for editing genomes in cancer research and therapy

The area of genetics has undergone a revolution because of the genome-editing technique known as CRISPR-Cas. It is a potent technique for understanding the functional arrangement of the genome and locating causative genetic variants as it enables targeted modification of any DNA sequence in the genome of any organism in vitro and in vivo. The employment of this technology for cancer research diagnosis and therapy has wide-ranging implications [33].

Mechanism of CRISPR-Cas9 in cancer treatment

**Figure 2:**
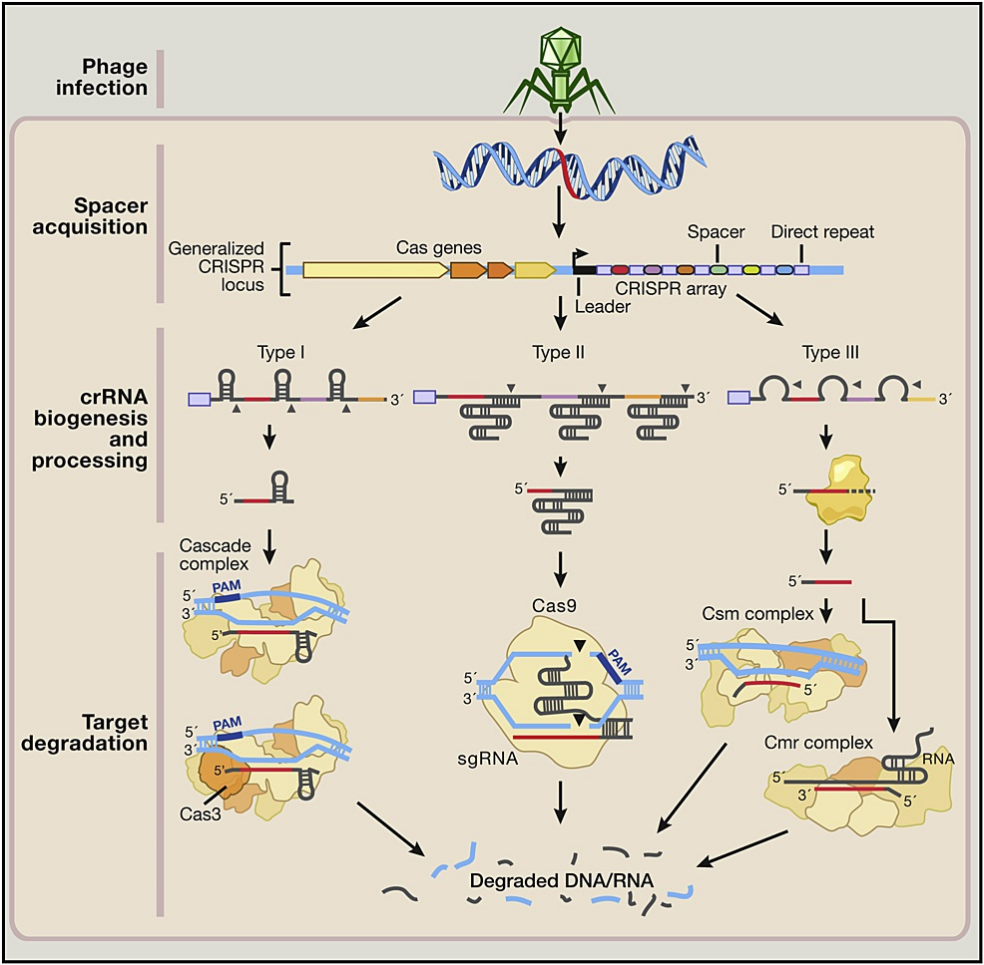
Natural mechanisms of microbial adaptive immune CRISPR systems: 1: phage infection, 2: spacer acquisition, and 3: cRNA biogenesis and processing. [33].

By removing cancer-causing genes and substituting healthy genes for them, the CRISPR-Cas9 system is an incredibly effective genome editing tool that is now being utilized to cure cancer. A designed single-guide RNA (sgRNA) and the Cas9 endonuclease comprise the system. With the aid of crRNA and tracrRNA, the sgRNA recognizes a particular target sequence and directs the Cas9 enzyme to break the DNA at that position. Based on the many bacterial and archaeal repeat sequences that have been identified as genes and their modes of action, the CRISPR-Cas systems have been divided into three primary kinds (I-III). Through the use of specialized Cas endonucleases, type I and III systems convert pre-crRNAs into crRNAs. Each crRNA complex containing multiple Cas proteins then recognizes and cleaves target sequences that are complementary to the crRNA. The type II system, in contrast, is the brain of the genome engineering tool because it uses fewer Cas enzymes [34,35]. While CRISPR-Cas9 can be delivered in vitro to a specific location and is very effective for cancers with single gene mutations, it becomes challenging for cancers with metastatic disease [34]. The CRISPR-Cas9 system’s injection procedure for snipping and inserting a gene into liver cells has been covered by Yin et al. [36] (Figure 2).

By removing cancer-causing genes and substituting healthy genes for them, the CRISPR-Cas9 system is an incredibly effective genome editing tool that is now being utilized to cure cancer. sgRNA and the Cas9 endonuclease make up the system. With the aid of crRNA and tracrRNA, the sgRNA recognizes a particular target sequence and directs the Cas9 enzyme to break the DNA at that position. Based on the many bacterial and archaeal repeat sequences that have been identified as genes and their modes of action, the CRISPR-Cas systems have been divided into three primary kinds (I-III). Through the use of specialized Cas endonucleases, type I and III systems convert pre-crRNAs into crRNAs. Each crRNA complex containing multiple Cas proteins then recognizes and cleaves target sequences that are complementary to the crRNA. The type II system, in contrast, is the brain of the genome engineering tool because it uses fewer Cas enzymes [34,35]. While CRISPR-Cas9 can be delivered in vitro to a specific location and is very effective for cancers with single gene mutations, it becomes challenging for cancers with metastatic disease [34]. The CRISPR-Cas9 system’s injection procedure for slicing and inserting a gene into liver cells was covered by Yin et al. [36]. With the aid of sgRNA, the CRISPR-Cas9 system from *Streptococcus pyogenes* is an RNA-guided DNA endonuclease that is targeted to a particular DNA sequence. The protospacer adjacent motif (PAM), which is commonly NGG or NAG, is located next to a particular target sequence with which the sgRNA base pairs. The Cas9 enzyme causes double-stranded breaks or nicks at the targeted areas, and either the non-homologous end joining (NHEJ) or homology-directed repair (HDR) pathway can be used to repair the damage. While HDR is a precise repair technique that uses homologous donor template DNA to repair DNA damage at the target site, NHEJ is an error-prone repair mechanism that causes heterogeneous insertions and deletions (indels) upon the joining of broken ends [34].

Advantages of CRISPR over conventional approaches

Over ZNFs and TALENs, the CRISPR/Cas9 system has several advantages. First, because CRISPR relies on ribonucleotide complex synthesis rather than DNA recognition, the target design process is less complicated, cheaper, and does not require time-consuming cloning stages. In addition, CRISPR is far more effective than ZFNs and TALENs and can target any particular DNA sequence in the genome. The host genome may be directly modified by injecting the RNA encoding the Cas protein, and numerous genes can be concurrently altered by infusing several different gRNAs. This procedure is excellent for GC-rich target regions as it is quicker than conventional techniques and is not impacted by DNA methylation [12,32].

## Conclusions

The discoveries of molecular biology have fundamentally changed the field of cancer research and therapy. This in-depth analysis has traveled through the complex realm of oncogenes, tumor suppressor genes, viral infections, and the various types of cancer-related gene classifications. In addition to shedding light on the genetic causes of cancer, molecular biology has also produced vital diagnostic and therapeutic tools. We have been able to unravel the genetic complexity of cancers using methods such as NGS, which has helped to inform tailored therapy approaches. Despite the emergence of several therapy options, one technology, the CRISPR-Cas9 system, remains at the cutting edge of innovation. With the potential for precise and tailored cancer therapy, this ground-breaking genome-editing technology offers patients everywhere fresh hope. Finally, the constant quest for molecular understanding has brought us one step closer to overcoming the enormous problem of cancer. Molecular biology has given us the tools to fight it in addition to illuminating the intricate details of its development. The future of cancer therapy shines brighter and brighter as we carry on with our exploration, discovery, and innovation.
